# Supplementary material for: Optical spin-orbit torque in heavy metal-ferromagnet heterostructures
Source: Nat Commun. 2020 Mar 20;11:1482. doi: 10.1038/s41467-020-15247-3 (PMC7083953; doi:10.1038/s41467-020-15247-3)
Supplement: Supplementary file 1 — Supplementary Information [file 41467_2020_15247_MOESM1_ESM.pdf]

Supplementary Information for

"Optical spin-orbit torque in heavy metal-ferromagnet heterostructures"

Gyung-Min Choi[1,2,3], Jung Hyun Oh[4], Dong-Kyu Lee[4], Seo-Won Lee[4], Kun Woo Kim[5], Mijin Lim[6], Byoung-Chul Min[3], Kyung-Jin Lee[4,7], and Hyun-Woo Lee[6]

[1]Department of Energy Science, Sungkyunkwan University, Suwon 16419, Korea

[2]Center for Integrated Nanostructure Physics, Institute for Basic Science (IBS), Suwon 16419, Korea

[3]Center for Spintronics, Korea Institute of Science and Technology, Seoul 02972, Korea

[4]Department of Materials Science and Engineering, Korea University, Seoul 02841, Korea

[5]Center for Theoretical Physics of Complex Systems, Institute for Basic Science (IBS), Daejeon 34051, Korea

[6]Department of Physics, Pohang University of Science and Technology, Pohang 37673, Korea

[7]KU-KIST Graduate School of Converging Science and Technology, Korea University, Seoul 02841, Korea

G.M.C. and J.H.O. equally contributed to this work.

Correspondence should be addressed to G.M.C. (gmchoi@skku.edu), K.J.L. (kj_lee@korea.ac.kr) and H.W.L. (hwl@postech.ac.kr)

Supplementary Note 1: Pump fluence dependence

We check the pump fluence dependence of the amplitudes of the OSOT in the sap/Co(10)/Pt(2) structure. The OSOT shows a linear dependence on the pump fluence (Supplementary Figure 1).

Supplementary Note 2: Probe polarization dependence at polar MOKE geometry

When there is a misalignment of the incidence angle of probe or sizable quadratic magneto-optical (MO) response, M_y or M_x component can be mixed with M_z component in a polar MOKE configuration [1]. To check the possible contribution from M_y or M_x component, we check the probe polarization dependence of the polar MOKE. The Kerr rotation by M_y or M_x component depends on the angle between probe polarization and magnetization (θ_{probe}), but the Kerr rotation by M_z component does not. The measured Kerr rotation is nearly constant with θ_{probe}, indicating that polar MOKE measures mostly M_z component (Supplementary Figure 2).

Supplementary Note 3: Measurement of M_y dynamics at longitudinal MOKE geometry

To measure the M_y dynamics, we inject the probe beam to the sample with an oblique angle, θ_0, in the *y*-*z* plane (Supplementary Figure 3). In this geometry, the measured Kerr rotation is a mixture of the polar Kerr rotation (θ_K^p), which is due to M_z dynamics, and the longitudinal Kerr rotation (θ_K^l), which is due to M_y dynamics. When the sample consists of a single magnetic layer whose thickness is much thicker than the optical penetration depth, the ratio between the θ_K^l and θ_K^p has a simple relationship of $\frac{\theta_K^l}{\theta_K^p} = \tan\theta_1$, where θ_1 is the angle of

refraction determined by θ_0 and refractive index of the magnetic layer [2]. When the sample consists of multilayers with a thickness thinner than the optical penetration depth, there is no simple relation between θ_K^l and θ_K^p. To distinguish θ_K^l and θ_K^p, we measure the Kerr rotation with an applied field of +*x* and –*x* directions. The sign of the M_z dynamics does not change with +*x* and –*x* directions of the applied field, but the sign of the M_y dynamics does. At a normal incidence angle, there is no difference in the Kerr rotation with +*x* and –*x* directions of the applied field (Supplementary Figure 4 (a)), whereas there is a clear difference at an oblique incidence angle (Supplementary Figure 4 (b)). The M_z and M_y dynamics can be obtained from the even and odd parts (with respect to +*x* and –*x* applied field) of the measurement at oblique incidence angle (Supplementary Figure 4 (c)). Fig. 2 (b) of the main text is obtained by normalizing the plot of Supplementary Figure 4 (a).

Supplementary Note 4: Effect of a film sequence

At polar MOKE geometry, the oscillation phase of the uniform precession of the Co magnetization remains unchanged regardless of whether the stacking sequence is sap/Pt/Co or sap/Co/Pt (Supplementary Figure 5). The magnitude of the Kerr rotation depends on the film sequence and leads to the different quantum efficiency (η) in Fig. 6 (a) on the main text.

Supplementary Note 5: Calculation of light absorption

We calculate the Poynting vector of pump light as it passes through the sample using a transfer matrix method. The decrease of the Poynting vector by each layer represents the amount of light absorption. The inset of Fig. 2 (c) of the main text shows the calculation results of the light absorption of Co and Pt in the sap/Co(x)/P(2) structure with refractive indexes of

1.76, 2.8 + *i* 4.9, and 2.5 + *i* 4.7 for sapphire, Pt, and Co, respectively [3-5]. For other structures of sap/HM/Co/MgO or sap/HM/Cu/Co/MgO, where HM is Ta, W, Pd, or Pt, we use additional refractive indexes of 3.6 + *i* 2.8, 3.6 + *i* 2.8, 2.1 + *i* 4.9, and 0.3 + *i* 4.9 for Ta, W, Pd, and Cu, respectively [4, 5]. In Supplementary Table I, we show the calculation results of the light absorption of HM in sap/HM(5)/Co(3)/MgO(3) and sap/HM(5)/Cu(2)/Co(3)/MgO(3) structures.

Supplementary Note 6: Static Kerr rotation

To convert the dynamic Kerr rotation during the time-resolved MOKE experiment to the fractional change of magnetization, we use as reference the static Kerr rotation that corresponds to the saturation magnetization. The static Kerr rotation depends on the thickness of Co and Pt in the sap/Co/Pt structures (Supplementary Figure 6). At a fixed Pt thickness, the static Kerr rotation increases with the Co thickness up to 10 nm, decreases up to 30 nm, and saturates after 30 nm (Supplementary Figure 6 (a)). The peak of the static Kerr rotation at the Co thickness of 10 nm is caused by an additional contribution from the Faraday rotation [5]. At a fixed Co thickness, the static Kerr rotation gradually decreases with the Pt thickness ((Supplementary Figure 6 (b)). In addition, the static Kerr rotation of Co depends on the materials of the underlayer of HM ((Supplementary Figure 6 (c)).

Supplementary Note 7: Modeling for charge current

Assuming that the *E*-field at the Pt/Cu interface is strong enough, we calculate the photo-induced charge transport in the Pt(5)/Cu(x)/Co(3) structure using SPICE simulation. Since the charge current flows along the out-of-plane direction in metallic multilayers on top

of the insulating substrate, it is subject to the open circuit situation. With a homogeneous metal at the open circuit, any forward charge current (J_c) will be quickly canceled out by the backward current. However, when an electric field at the interface is strong enough, the backward current should be blocked. The circuit diagram for the simulation is shown in Supplementary Figure 7 (a). The photocurrent is modeled as a current source that generates a forward current (J_c). The J_c at the Pt/Cu interface is a Gaussian pulse with FWHM of 1.1 ps, and its magnitude is set by $\int J_{\mathrm{c}}\, dt = \frac{q}{\hbar\omega} F_{\mathrm{in}} A_{\mathrm{Pt}}$. The effect of the electric field at the Pt/Cu interface is modeled as a diode, which blocks a backward current. The threshold voltage of the diode is set to 0.7 V. The capacitances of Cu and Co are determined from the electronic density of states at Fermi level (N_F) and thicknesses (d) as $C = N_F \cdot d$. The resistance of Cu and Co are determined from bulk electric resistivity and interfacial resistance. But as long as they are small enough, it turns out that they do not affect the simulation result. The charge accumulation ($Q_c = \int J_{\mathrm{c}}\, dt$) at the Co capacitance decreases with increasing Cu thickness with an exponential length scale of 20~30 nm (Supplementary Figure 7 (b)). We also calculate the charge accumulation on Co with a relative ratio of the electronic capacitance of Co and Cu. When we ignore the resistance effect and assume no backward current, charge accumulation Co (Q_{Co}) will be determined by

$$Q_{\mathrm{Co}} = Q_0 \frac{N_{\mathrm{F\_Co}} d_{\mathrm{Co}}}{N_{\mathrm{F\_Cu}} d_{\mathrm{Cu}} + N_{\mathrm{F\_Co}} d_{\mathrm{Co}}}, \quad (1)$$

where Q_0 is the time integration of J_c. When the d_{Co} is fixed, the Q_{Co} decrease with increasing d_{Cu}, and the calculated length scale of the decaying is 20~30 nm, which is the same as the SPICE simulation (Supplementary Figure 7 (b)).

The length scale of 20~30 nm of the charge current at open circuit might look too long considering the short time scale for the dielectric relaxation time in metal, $\tau_{\mathrm{dr}} = \frac{\epsilon}{\sigma} \approx$

10^{-18} sec, where ϵ is permittivity and σ is electrical conductivity [7]. However, we would like to point out that having short τ_{dr} does not imply a short characteristic length scale of charge current transport. To demonstrate this point, one begins with the local charge conservation equation,

$$\frac{\partial \rho}{\partial t} + \nabla \cdot \mathbf{J} = 0, \tag{2}$$

where ρ is the charge density and $\mathbf{J}$ is the charge current density. One then combines this equation with the Ohm's law $\mathbf{J}=\boldsymbol{\sigma}\cdot\mathbf{E}$, where $\mathbf{E}$ is the electric field, and the Gauss law $\nabla \cdot \mathbf{E} = \rho/\epsilon$ to obtain the approximate equation,

$$\frac{\partial \rho}{\partial t} + \frac{\rho}{\tau_{\mathrm{dr}}} = 0. \tag{3}$$

The solution of this approximate equation is given by $\rho(\mathbf{r}, t) = \rho(\mathbf{r}, t = 0)\exp(-t/\tau_{dr})$, which implies that the initial charge density at $t = 0$ decays exponentially fast in time with the characteristic time scale τ_{dr}. We are now ready to discuss the length scale. For concreteness, one considers a metallic thin film (shown below) and assumes $\rho(\mathbf{r}, t = 0)$ to be spatially homogeneous within the film, $\rho(\mathbf{r}, t = 0) = \rho_0$ (this assumption is not crucial, however).

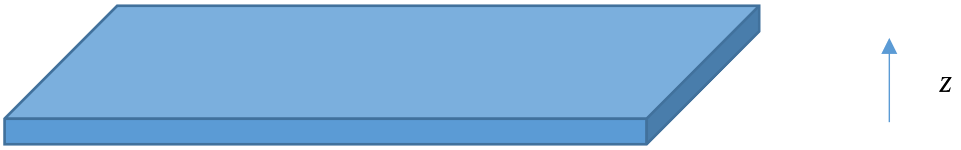

For this film geometry, one then uses the Gauss law to calculate the electric field $\mathbf{E}$ from the above solution $\rho(\mathbf{r}, t)$ to obtain $\mathbf{E}(\mathbf{r}, t) = \hat{\mathbf{z}}\left(\frac{z\rho_0}{\epsilon}\right)\exp\left(-\frac{t}{\tau_{\mathrm{dr}}}\right) + \text{constant}$. Together with the Ohm's law, one obtains

$$\mathbf{J}(\mathbf{r},t) = \hat{\mathbf{z}} z \frac{\rho_0}{\tau_{\mathrm{dr}}} \exp\left(-\frac{t}{\tau_{\mathrm{dr}}}\right) + \mathrm{constant}, \tag{4}$$

where z is the coordinate along the thickness direction and $\hat{\mathbf{z}}$ is the unit vector along the z direction. Note that $\mathbf{J}(\mathbf{r},t)$ does grow in space (proportional to z), implying that the characteristic length scale is *long-ranged*. This long-rangedness persists even for spatially localized $\rho(\mathbf{r},t=0)$, say, $\rho(\mathbf{r},t=0) = A\delta(z)$. For this initial condition, one obtains

$$\mathbf{J}(\mathbf{r},t) = \hat{\mathbf{z}} \mathrm{sgn}(z) \frac{A}{2\tau_{\mathrm{dr}}} \exp\left(-\frac{t}{\tau_{\mathrm{dr}}}\right), \tag{5}$$

where $\mathrm{sgn}(z)$ is the Heaviside step function. Note that $\mathbf{J}(\mathbf{r},t)$ for this choice of $\rho(\mathbf{r},t=0)$ is also spatially long-ranged. This result for $\mathbf{J}(\mathbf{r},t)$ actually implies that when a local charge density $\rho(\mathbf{r},t=0) = A\delta(z)$ (assume $A > 0$) is introduced by an external perturbation at time $t = 0$ at the mid-plane ($z = 0$) of the film, the initial charge relaxes by generating $\mathbf{J}(\mathbf{r},t)$ [toward $+\hat{\mathbf{z}}$ for $\mathrm{z} > 0$ and toward $-\hat{\mathbf{z}}$ for $\mathrm{z} < 0$] over the *entire* thickness of the film. This charge current density is maintained for the time of the order of τ_{dr} and after this time, the initial charge is entirely transported to the upper and lower surfaces of the film and piled up there due to the open boundary condition. This verifies that even when the characteristic time scale τ_{dr} is very short, $\mathbf{J}(\mathbf{r},t)$ within this time interval can be extended over long distances. In the approximation presented above, the characteristic length scale of $\mathbf{J}(\mathbf{r},t)$ is bounded only by the film thickness.

Supplementary Table I: The calculation of the light absorption (abs) of HM, where HM is Ta, W, Pd, or Pt, of sap/HM(5)/Co(3)/MgO(3) and sap/HM/Cu(2)/Co(3)/MgO(3) structures. The magnitude of the initial Poynting vector of pump light, which is incident on the sapphire substrate, is set to be one.

structure	Sap/HM(5)/Co(3)/MgO(3)				Sap/HM(5)/Cu(2)/Co(3)/MgO(3)			
HM	Ta	W	Pd	Pt	Ta	W	Pd	Pt
abs	0.336	0.336	0.316	0.378	0.330	0.330	0.298	0.361

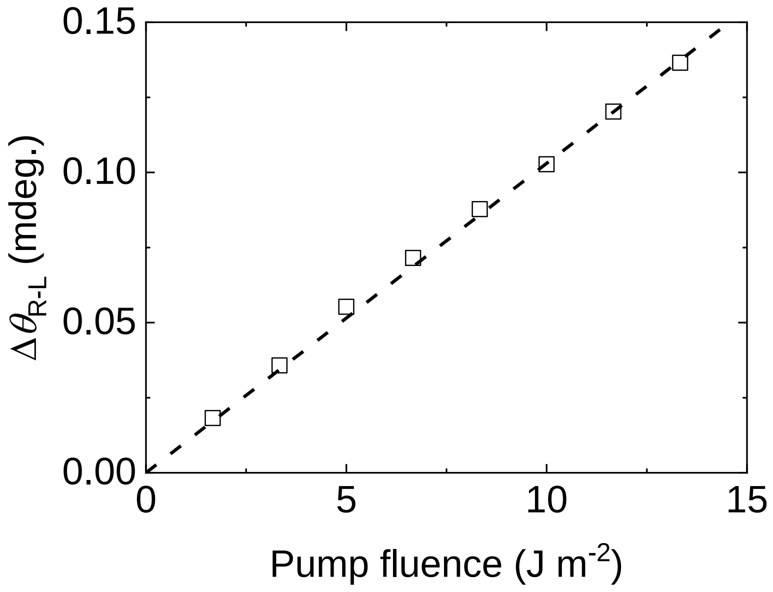

Supplementary Figure 1: Pump fluence dependence of the amplitudes of the OSOT (black squares). The dashed line is a linear fit to data. Data are taken by the difference between right and left circularly polarized pump pulses ($\Delta\theta_{R\text{-}L}$).

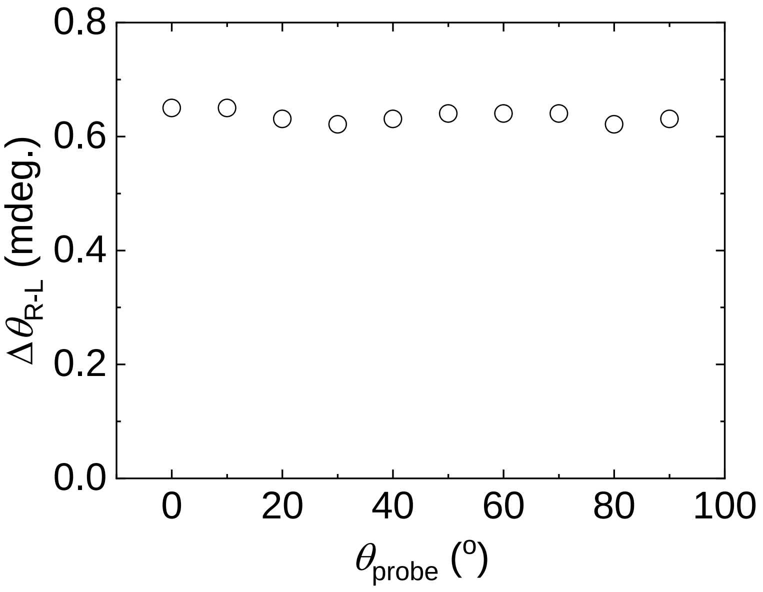

Supplementary Figure 2: Probe polarization dependence of polar MOKE. The Kerr rotation with a normal incidence angle of the probe with the Pt(6)/Co(3) sample is measured with different angles between probe polarization and Co magnetization. Data are taken by the difference between right and left circularly polarized pump pulses ($\Delta\theta_{R-L}$).

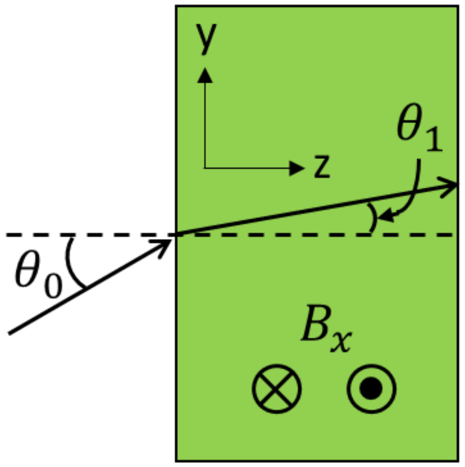

Supplementary Figure 3: Measurement of longitudinal MOKE. The probe light is incident on the sample with an oblique angle of θ_0 in the *y*-*z* plane. To distinguish the Kerr rotation due to M_z dynamics and that due to M_y dynamics, measurements are performed with an external magnetic field (B_x) along the +*x* and –*x* directions, simultaneously.

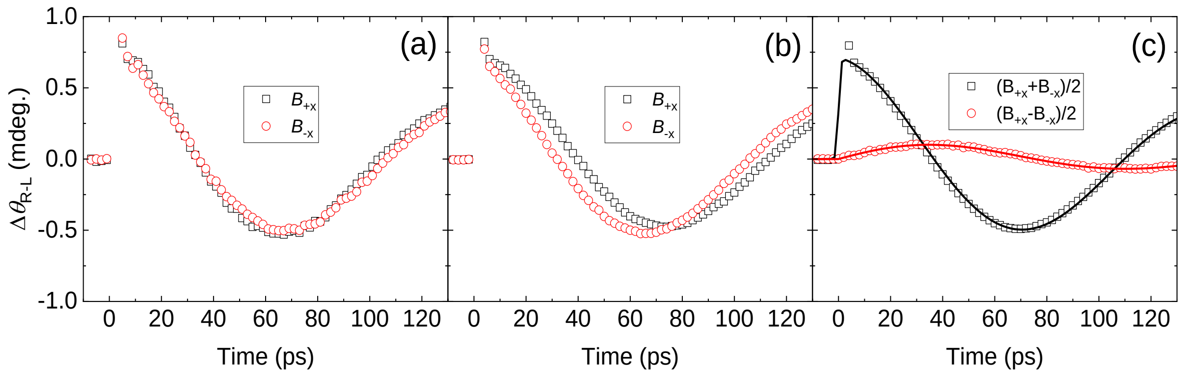

Supplementary Figure 4: Distinguishing M_z and M_y dynamics. The helicity-driven Kerr rotation ($\Delta\theta_{R-L}$) of the sap/Pt(5)/Co(3)/MgO(3) sample at (a) polar geometry and (b) longitudinal geometry. An external magnetic field of 0.06 T is applied along the +*x* (black squares) and –*x* (red circles) directions. (c) The M_z and M_y dynamics are obtained from even and odd part, respectively, of the raw data of (b). The black and red lines are fitting with damped-cosine and damped-sine functions, respectively.

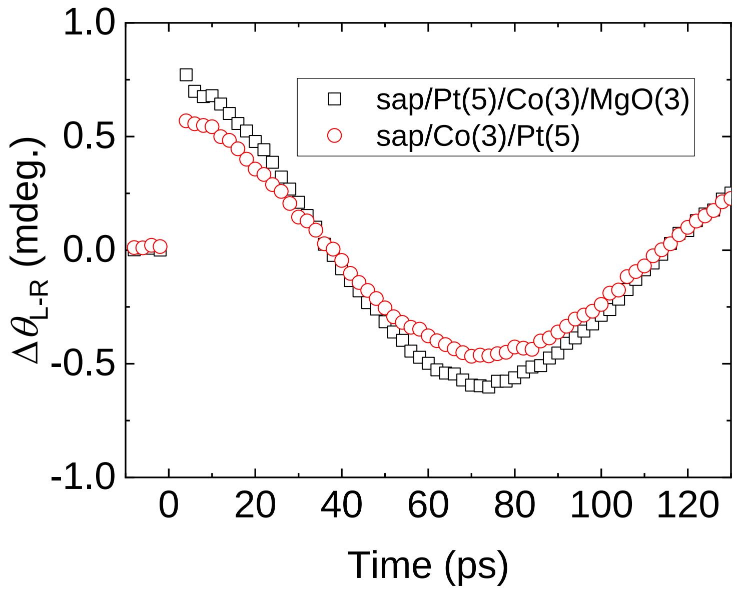

Supplementary Figure 5: The effect of the film stacking sequence on the M_z dynamics. The helicity-driven Kerr rotation ($\Delta\theta_{R\text{-}L}$) of the sap/Pt(5)/Co(3)/MgO(3) (black squares) and sap/Co(3)/Pt(5) (red circles) sample at polar MOKE geometry.

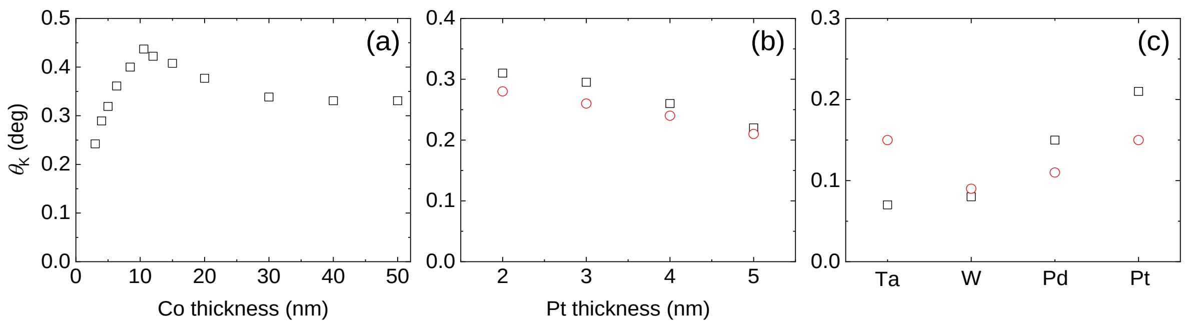

Supplementary Figure 6: Static Kerr rotation for (a) sap/Co(d_{Co})/Pt(2) (black circles), (b) sap/Co(3)/Pt(d_{Pt}) (black squares) and sap/Pt(d_{Pt})/Co(3) (red circles), and (c) sap/HM(5)/Co(3) (black circles) and sap/HM(5)/Cu(2)/Co(3) (red circles) samples, where HM is Ta, W, Pd, and Pt.

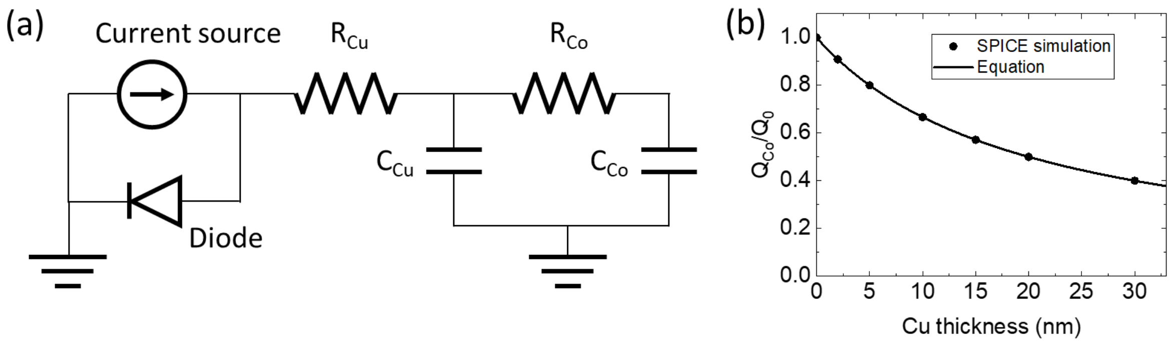

Supplementary Figure 7: SPICE modeling for charge transport. (a) The built-in potential develops an electric field at the interface with a typical width (w) of ≈1 nm. The electric field at the Pt/Cu interface induces a forward charge current (J_c) of photo-excited carriers but blocks the backward J_c. (b) Circuit diagram for the simulation of charge current. (c) The simulation result of the charge accumulation on the Co capacitor (Q_{Co}) normalized by total charge ($Q_o = \int J_c dt$) (black circles). The solid line is the calculation with equation (1) of Supplementary Note 7.

Supplementary References

1. Buchmeier, M., Schreiber, R., Bürgler, & Schneider, C. M. Thickness dependence of linear and quadratic magneto-optical Kerr effects in ultrathin Fe(001) films. *Phys. Rev. B* **79**, 064402 (2009).

2. You, C.-Y. & Shin, S.-C. Generalized analytic formulae for magneto-optical Kerr effects. *J. Appl. Phys.* **84**, 541 (1998).

3. *CRC Handbook of chemistry and physics*. 94th edition. (CRC press, 2013).

4. Johnson, P. B. & Christy, R. W. Optical constants of transition metals: Ti, V, Cr, Mn, Fe, Co, Ni, and Pd. *Phys. Rev. B* **9**, 5056-5070 (1974).

5. Rakić, A. D., Djurišić, A. B., Elazar, J. M., & Majewski, M. L. Optical properties of metallic films for vertical-cavity optoelectronic devices. *Appl. Opt.* **37**, 5271-5283 (1998).

6. Moog, E. R., Liu, C., Bader, S. D., & Zak, J., Thickness and polarization dependence of the magneto-optic signal from ultrathin ferromagnetic films, *Phys. Rev. B* **39**, 6949-6956 (1989).

7. Kimling, J. & Cahill, D. G. Spin diffusion induced by pulsed-laser heating and the role of spin heat accumulation. *Phys. Rev. B* **95**, 014402 (2017).
